# Supplementary material for: Single-electron charge transfer into putative Majorana and trivial modes in individual vortices
Source: Nat Commun. 2023 Jun 8;14:3341. doi: 10.1038/s41467-023-39109-w (PMC10247703; doi:10.1038/s41467-023-39109-w)
Supplement: Supplementary file 2 — Supplementary Information [file 41467_2023_39109_MOESM2_ESM.pdf]

Supplementary Information for

Single-electron charge transfer into putative Majorana and trivial modes in individual vortices

*Jian-Feng Ge,[1] Koen M. Bastiaans,[1, 2] Damianos Chatzopoulos,[1] Doohee Cho,[3] Willem O. Tromp,[1] Tjerk Benschop,[1] Jiasen Niu,[1] Genda Gu,[4] Milan P. Allan[1]**

[1] Leiden Institute of Physics, Leiden University, 2333 CA Leiden, The Netherlands
[2] Department of Quantum Nanoscience, Kavli Institute of Nanoscience, Delft University of Technology, 2628 CJ Delft, The Netherlands
[3] Department of Physics, Yonsei University, Seoul 03722, Republic of Korea
[4] Condensed Matter Physics and Materials Science Department, Brookhaven National Laboratory, Upton, NY, 11973, USA

*Corresponding author. Email: allan@physics.leidenuniv.nl

Supplementary Note 1: Characterization of the superconducting tip

We decorate a mechanically ground Pt-Ir tip with Pb microcrystals by indenting into a Pb(111) single crystal, cleaned beforehand by standard Ar^+ sputtering cycles, until a superconductor-insulator-superconductor (SIS) tunnel junction is established (Supplementary Fig. 1a). The energy resolution is estimated to be 0.25 meV from the full width at half maxima of the sharp coherence peaks in Supplementary Fig. 1a.

Then we perform tunneling spectroscopy with this tip on a clean Au(111) surface at various magnetic fields, as shown in Supplementary Fig. 1b. The differential conductance measured by tunneling spectroscopy is expressed by

$$\frac{\mathrm{d}I(V)}{\mathrm{d}V} \propto \int \mathrm{d}E\ N_\mathrm{s}(E)\frac{\partial}{\partial V}\{N_\mathrm{t}(E+eV)[f(E,T)-f(E+eV,T)]\}, \tag{1}$$

where E is energy, N_s (N_t) is the density of states (DOS) in the sample (tip), and $f(E, T) = [1+\exp(E/k_\mathrm{B}T)]^{-1}$ is the Fermi function (k_B being the Boltzmann constant). We model the tip DOS by the Dynes function[1]

$$N_\mathrm{t}(E,V,\Gamma) = \mathrm{Re}\left[\frac{E+i\Gamma}{(E+i\Gamma)^2-\Delta_\mathrm{t}^2}\right], \tag{2}$$

where Δ_t is the superconducting energy gap of the tip and Γ is the phenomenological broadening parameter (without thermal broadening). The values for Δ_t and Γ are extracted by fitting each spectrum to Supplementary Equation 1, assuming a constant N_s for Au(111) in the energy range from -10 meV to +10 meV. The fit results are summarized in Supplementary Fig. 1c, showing a critical field of 0.7 T, about 7 times larger than that of the bulk Pb.[2]

Supplementary Note 2: Deconvolution of the conductance spectra taken with a superconducting tip

The consequence of using a superconducting tip with an energy gap Δ_t, as illustrated by Supplementary Fig. 2a, is the resonance tunneling when either of the gap edges of the tip DOS

aligns with the zero-energy state (ZES). Thus, the bound state appears as peaks at $\pm\Delta_t$ in the measured differential conductance spectra. Deconvolution of a differential conductance spectrum is necessary to recover the appearance of the bound state at zero energy in the sample DOS, the same as what one would expect for a spectrum taken with a normal-metal tip.[3, 4] We follow the deconvolution algorithm described in Refs. [5, 6] to extract the sample DOS N_s in Supplementary Equation 1. We use the fit results at B = 0.1 T in Supplementary Fig. 1c for the tip DOS N_t in the deconvolution.

To determine the energy of the vortex bound states in $FeTe_{0.55}Se_{0.45}$, in Supplementary Fig. 2b, c we stack the raw and deconvoluted spectra for the line cut in Fig. 3c, f, respectively. We fit each peak (at $\pm\Delta_t$ for Supplementary Fig. 2b and at 0 meV for Supplementary Fig. 2c) with a Lorentzian function, and plot the peak energy as a function of the position along the line in Supplementary Fig. 2d. We note an additional broadening of the coherence peaks and the zero-bias peak in the deconvoluted DOS (Fig. 3e) compared to the peaks in the raw spectrum in Fig. 3b, due to the need in the deconvolution algorithm to remove oscillatory errors (we chose the optimized value for the control parameter γ = 5.0 defined in Ref. [5]). Nevertheless, the peak center locates at 0 ± 50 μeV, confirming them as zero-energy states. On the other hand, from the amplitudes of the Lorentzian fit (Supplementary Fig. 2e), we find the zero-energy state has roughly an exponential decay in DOS, with a decay length of ~ 4.0 nm on both sides of the core center.

Supplementary Note 3: Differential conductance and noise spectroscopy on different vortices

We present the full dataset of both differential conductance and noise spectroscopy performed on all vortices in Supplementary Figs. 3 (for $NbSe_2$) and 4 (for $FeTe_{0.55}Se_{0.45}$), except the ones shown in Figs. 2 to 4. For each material, all vortices exhibit similar behavior as illustrated in the main text, especially a zero-bias peak in the DOS after deconvolution and 1*e*-noise on vortex. We show in Supplementary Figs.5a and b the extracted effective charge q^* as a function of bias energy for the noise spectra in Figs. 4a and c, respectively. The error bars of q^* in Fig. 4b (4d) are extracted from Supplementary Figs.5a, 3h, 3p (Supplementary Figs. 5b, 4h, 4p), within an energy window of 0.2 meV centered at $\pm\Delta_t$. A bigger error in q^* for vortex #2 (and #3) in Fig. 4d is due to a higher junction resistance of 5 MOhm (and 10 MOhm) used for the noise spectroscopy measurements, leading to reduced absolute values of tunnel current and its noise.

Supplementary Note 4: Scanning noise spectroscopy at zero field around a YSR impurity

The results of our measurements on $FeTe_{0.55}Se_{0.45}$ at zero magnetic field are shown in Supplementary Fig. 6. Here we observe the Yu-Shiba-Rusinov (YSR) bound states as a ring in the differential conductance map (Supplementary Fig. 6b). The YSR states lead to a negative differential conductance in the spectrum (Supplementary Fig. 6d) because of the convolution of a sharp in-gap resonance peak and superconducting tip DOS as we observed in a previous study.[6] The noise spectrum measured *away* from the impurity site (Supplementary Fig. 6e) shows clear transitions from q^* = 1*e* line to q^* = 2*e* line with onsets at $\pm(\Delta_t + \Delta_s)$, indicating a dominating Andreev reflection inside the gap. This noise behavior is similar to what we observed before on Pb(111) surface with a superconducting tip.[7] We extract the effective charge q^* in Supplementary Fig. 6f by numerically solving Eq. 2 in the main text. We observe a narrower step of q^* from 1*e* outside the gap to 1.97*e* at $E = \pm\Delta_t$, compared to the broader

transition from 1e to a plateau of 1.3e~1.6e in Supplementary Fig. 5. The value of q^* so close to 2e in Supplementary Fig. 6f indicates the tunneling current originates purely from Andreev reflection in the SIS junction, whereas q^* short of 2e in Supplementary Fig. 5 off vortex implies that the tunneling process is not purely Andreev reflection, and that a contribution from 1e-charge tunneling coexists.

A previous study[8] demonstrated, by both theory and experiment, that Andreev process dominates single-particle tunneling into YSR states in the strong tunneling limit $\Gamma_1 << \Gamma_t$, where Γ_t is the tunneling rate and Γ_1 is the threshold rate for quasiparticles to be excited into the continuum. For our experiment on $FeTe_{0.55}Se_{0.45}$, we estimate Γ_1 = 1.1 μeV from Eq. (S49) of Ref. [8], using Δ_s = 1.5 meV, T = 2.3 K, and the YSR energy of 0.3 meV. This yields a threshold current of ~ 90 pA from Eq. (S65) of Ref. [8]. We expect that, in this limit, the effective charge when tunneling into YSR states is identical to that into the bare superconductor at a bias energy inside the gap.

We have verified in the strong tunneling limit (with a current of 800 pA) that the YSR state does not cause a spatial difference in shot noise, as shown by the noise map in Supplementary Fig. 6c, compared to the prominent ring feature in the differential conductance map taken in the same field of view and at the same bias voltage (Supplementary Fig. 6b). Therefore, we exclude YSR states as the origin of the zero-energy bound states in the vortex cores of $FeTe_{0.55}Se_{0.45}$.

Note that there is no contradiction between Ref. [9] and our data for YSR states. For tunneling into the bare superconductor $NbSe_2$, Ref. [9] measures 1e noise outside the gap but lacks of noise data inside the gap because of the low current (~20 pA), for which they could not detect the corresponding shot noise accurately. In principle, considering a normal tip used in this case, inside the gap q^* increases gradually to 2e [10]. For tunneling into YSR states, Ref. [9] elucidates that Andreev reflection requires particle-hole symmetric resonances. The smaller of the asymmetric resonances leads to a deficiency of providing particle (or hole) for the Andreev (2e) process, so that tunneling into the excess hole (or particle) component in the larger resonance has to be mediated by an inelastic quasiparticle relaxation process. In our experiment this inelastic quasiparticle relaxation (1e) process is strongly suppressed because the YSR states in $FeTe_{0.55}Se_{0.45}$ are almost symmetric in amplitude and our noise measurements were carried out in the strong tunneling regime. In summary, both our data and Ref. [9] reach the conclusion that tunneling into YSR states, when inelastic quasiparticle relaxation can be neglected, gives 2e noise.

Supplementary Note 5: The effective charge when single-particle and Andreev processes both contribute

In this section, we calculate, based on an empirical model, the effective charge when both Andreev reflection and quasiparticle of 1e tunneling contribute to the total current. Away from the vortex, the tunnel junction is similar to an SIS junction, as shown by comparing the spectra in Fig. 2b and Fig. 3b and the zero-field spectrum in Supplementary Fig. 6d. Especially at the bias energy $E = \pm\Delta_t$, the deconvolution yields a vanishing density of states of the sample (Figs. 2e and 3e). Therefore, the tunneling process for this tunnel junction is expected to be dominated by Andreev reflection that transfers a charge of 2e per event. We then introduce a fraction of the 1e-charge tunneling process,[11] which contributes to current and noise but has no correlation with those of the Andreev process. For a given tunneling transparency $\tau << 1$, the current contributions for the single-particle processes (I_{1e}) and the Andreev processes (I_{2e}) are proportional to τ and τ^2, respectively,[12]

$$I_{ne} \propto n\tau^n/4^{n-1},\ n = 1, 2. \tag{S3}$$

The prefactors are related to the integrated density of states, and here we assume an empirical prefactor y for quasiparticle contribution I_{1e} and 1-y for I_{2e} to have a conserved total integrated density of states. The total current is $I = I_{1e} + I_{2e}$. As I_{1e} and I_{2e} are assumed to be independent, the total current noise is the sum of both contributions,

$$S = 2eI_{1e}\coth(eV/2k_BT) + 2\cdot 2e\cdot I_{2e}\coth(2eV/2k_BT), \tag{S4}$$

where the double-charge ($2e$) transfer is taken into account in the Andreev contribution (the second term). Then we extract numerically the (total) effective charge q^* by Eq. 2.

Supplementary Figure 7 plots q^* as a function of the fraction of quasiparticle contribution for different junction resistance we used in noise measurements. When $I_{1e}/I = 0$, i.e., no single-particle process contributes, $q^* = 2e$ as expected from purely Andreev reflection. Conversely when $I_{1e}/I = 100\%$, only single-particle process contributes, yielding $q^* = 1e$. For values of I_{1e}/I in between 0 and 100%, we find a quick reduction of q^* even when a very small fraction of quasiparticle contribution exists (note the logarithmic scale of the horizontal axis). For example, for R_J = 2.5 MOhm, 0.02% of quasiparticle contribution reduces q^* to 1.92e, while 3.3% of quasiparticle contribution reduces already reduces q^* to 1.07e.

Supplementary Note 6: Transparency of the tunnel junction during noise spectroscopy

In differential conductance (Figs. 2 and 3) and noise (Fig. 4) spectroscopy, we use different setup conditions in terms of feedback control of the tip. Specifically, in differential conductance measurements, as the protocols are conventionally applied, feedback is disabled during voltage sweeps (spectroscopy). However, in noise spectroscopy, in order to have optimal junction stability, we enable a slow feedback to maintain a constant junction resistance (i.e., changing the bias voltage and current setpoint for each point in a sweep), except at the zero-bias point where feedback has to be disabled. As already presented in Fig. 2b, the differential conductance of an SIS junction varies by an order of magnitude during a sweep, the transparency of the junction, if feedback is enabled, could also vary considerably. The transparency τ, which is assumed to be in the $\tau << 1$ limit for current noise expressions in the main text, has a significant influence on the resulted noise when it becomes comparable to unity.[9] In Supplementary Fig. 8a we compare the differential conductance taken with feedback disabled and enabled, at different junction resistance (thus the setup τ). While outside the gap the conductance measured both ways is almost identical, a drastic difference in conductance develops in the gap because of a vanishing quasiparticle density of states. The ratio of the feedback-on conductance over the feedback-off conductance g_{on}/g_{off} indicates the enhancement of transparency from $\tau = (R_JG_0)^{-1}$ from an Ohmic current-voltage relation, where $G_0 = 2e^2/h$ = 77.5 μS is the conductance quantum (h being the Planck constant). In fact, the conductance ratio g_{on}/g_{off} is barely dependent on R_J, as shown in Supplementary Fig. 8b. To estimate the highest τ inside the gap throughout our noise measurements around vortices, we approximate $\tau = (R_JG_0)^{-1}$ for the setup bias, where g_{on}/g_{off} is close to 1. For simplicity, we model the sample DOS N_s also by the Dynes function (Supplementary Equation 2) and calculate the feedback-off conductance g_{off} by Supplementary Equation 1. In this case, the conductance ratio g_{on}/g_{off} is equal to the current ratio I_{on}/I_{off}. Therefore, we first integrate g_{off} to get I_{off}, and then we obtain the ratio $g_{on}/g_{off} = I_{on}/I_{off} = VR_J/I_{off}$. Using this simple model, we can simulate the ratio g_{on}/g_{off} (the green line in Supplementary Fig. 8b), in good agreement with the experimental

results. From Supplementary Fig. 8 we have confirmed that in our measurement conditions, enabling feedback has a marginal effect on the noise, as the transparency τ stays below 0.16, which is still in the $\tau << 1$ limit.

Supplementary Note 7: Different dispersions of CdGM states in $NbSe_2$ and $FeTe_{0.55}Se_{0.45}$

For a conventional s-wave BCS superconductor, CdGM states have been extensively studied theoretically (e.g., Ref. S11): the CdGM states have an increasing angular momentum when moving a distance r away from the vortex core. As a consequence, the majority of the CdGM states that contributes to the differential conductance have an energy E_p approximately proportional to $k_F \cdot r$, where k_F is the Fermi wavevector. In addition, at E_p the differential conductance maximum decays exponentially in r on a length scale of coherence length ξ. Therefore, the dispersion profile of CdGM states depends crucially on two material parameters k_F and ξ (see Supplementary Table 1 for their values of $NbSe_2$ and $FeTe_{1-x}Se_x$). For $NbSe_2$, both parameters are larger, and the dispersion is measurable by STM. However, for $FeTe_{1-x}Se_x$, k_F is one order of magnitude smaller, so E_p changes much more slowly with r; meanwhile ξ is also smaller, resulting in a vanishing amplitude in differential conductance before E_p changes significantly. Therefore, observation of dispersing states indicates a CdGM origin, but non-dispersing states cannot exclude a CdGM origin.

Supplementary Table 1. Fermi wavevector and coherence length of $NbSe_2$ and $FeTe_{1-x}Se_x$.

Material	$NbSe_2$	$FeTe_{1-x}Se_x$
Fermi wavevector k_F	0.5~1 Å^{-1} [12]	0.07~0.12 Å^{-1} [13]
Coherence length ξ	12 nm [14]	3 nm [15]

Supplementary References

[1] R. C. Dynes, V. Narayanamurti, and J. P. Garno, "Direct Measurement of Quasiparticle-Lifetime Broadening in a Strong-Coupled Superconductor," *Phys. Rev. Lett.* **41**, 1509–1512 (1978). http://dx.doi.org/10.1103/PhysRevLett.41.1509 .

[2] H. Suderow, I. Guillamón, J. G. Rodrigo, and S. Vieira, "Imaging superconducting vortex cores and lattices with a scanning tunneling microscope," *Supercond. Sci. Technol.* **27**, 063001 (2014). http://dx.doi.org/10.1088/0953-2048/27/6/063001 .

[3] D. Wang, L. Kong, P. Fan, H. Chen, S. Zhu, W. Liu, L. Cao, Y. Sun, S. Du, J. Schneeloch, R. Zhong, G. Gu, L. Fu, H. Ding, and H.-J. Gao, "Evidence for Majorana bound states in an iron-based superconductor," *Science* **362**, 333–335 (2018). http://dx.doi.org/10.1126/science.aao1797 .

[4] S. Zhu, L. Kong, L. Cao, H. Chen, M. Papaj, S. Du, Y. Xing, W. Liu, D. Wang, C. Shen, F. Yang, J. Schneeloch, R. Zhong, G. Gu, L. Fu, Y.-Y. Zhang, H. Ding, and H.-J. Gao, "Nearly quantized conductance plateau of vortex zero mode in an iron-based superconductor," *Science* **367**, 189–192 (2020). http://dx.doi.org/10.1126/science.aax0274 .

[5] A. Palacio-Morales, E. Mascot, S. Cocklin, H. Kim, S. Rachel, D. K. Morr, and R. Wiesendanger, "Atomic-scale interface engineering of Majorana edge modes in a 2D magnet-superconductor hybrid system," *Sci. Adv.* **5**, eaav6600 (2019). http://dx.doi.org/10.1126/sciadv.aav6600 .

[6] D. Chatzopoulos, D. Cho, K. M. Bastiaans, G. O. Steffensen, D. Bouwmeester, A. Akbari, G. Gu, J. Paaske, B. M. Andersen, and M. P. Allan, "Spatially dispersing Yu-Shiba-Rusinov states in the unconventional superconductor FeTe0.55Se0.45," *Nat Commun* **12**, 298 (2021). http://dx.doi.org/10.1038/s41467-020-20529-x .

[7] K. M. Bastiaans, D. Cho, D. Chatzopoulos, M. Leeuwenhoek, C. Koks, and M. P. Allan, "Imaging doubled shot noise in a Josephson scanning tunneling microscope," *Phys. Rev. B* **100**, 104506 (2019). http://dx.doi.org/10.1103/PhysRevB.100.104506 .

[8] M. Ruby, F. Pientka, Y. Peng, F. von Oppen, B. W. Heinrich, and K. J. Franke, "Tunneling Processes into Localized Subgap States in Superconductors," *Phys. Rev. Lett.* **115**, 087001 (2015). http://dx.doi.org/10.1103/PhysRevLett.115.087001 .

[9] U. Thupakula, V. Perrin, A. Palacio-Morales, L. Cario, M. Aprili, P. Simon, and F. Massee, "Coherent and Incoherent Tunneling into Yu-Shiba-Rusinov States Revealed by Atomic Scale Shot-Noise Spectroscopy," Phys. Rev. Lett. 128, 247001 (2022). http://dx.doi.org/10.1103/PhysRevLett.128.247001 .

[10] K. M. Bastiaans, D. Chatzopoulos, J.-F. Ge, D. Cho, W. O. Tromp, J. M. van Ruitenbeek, M. H. Fischer, P. J. de Visser, D. J. Thoen, E. F. C. Driessen, T. M. Klapwijk, and M. P. Allan, "Direct evidence for Cooper pairing without a spectral gap in a disordered superconductor above T c," Science 374, 608–611 (2021). http://dx.doi.org/10.1126/science.abe3987 .

[11] P. Zhou, L. Chen, Y. Liu, I. Sochnikov, A. T. Bollinger, M.-G. Han, Y. Zhu, X. He, I. Božović, and D. Natelson, "Electron pairing in the pseudogap state revealed by shot noise in copper oxide junctions," *Nature* **572**, 493–496 (2019). http://dx.doi.org/10.1038/s41586-019-1486-7 .

[12] J. C. Cuevas, A. Martín-Rodero, and A. L. Yeyati, "Shot Noise and Coherent Multiple Charge Transfer in Superconducting Quantum Point Contacts," *Phys. Rev. Lett.* **82**, 4086–4089 (1999). http://dx.doi.org/10.1103/PhysRevLett.82.4086 .

[13] F. Gygi and M. Schlüter, "Self-consistent electronic structure of a vortex line in a type-II superconductor," *Phys. Rev. B* **43**, 7609–7621 (1991). http://dx.doi.org/10.1103/PhysRevB.43.7609 .

[14] D. J. Rahn, S. Hellmann, M. Kalläne, C. Sohrt, T. K. Kim, L. Kipp, and K. Rossnagel, "Gaps and kinks in the electronic structure of the superconductor 2H-$NbSe_2$ from angle-resolved photoemission at 1 K," *Phys. Rev. B* **85**, 224532 (2012). http://dx.doi.org/10.1103/PhysRevB.85.224532 .

[15] M. Chen, X. Chen, H. Yang, Z. Du, X. Zhu, E. Wang, and H.-H. Wen, "Discrete energy levels of Caroli-de Gennes-Matricon states in quantum limit in FeTe0.55Se0.45," *Nat Commun* **9**, 970 (2018). http://dx.doi.org/10.1038/s41467-018-03404-8 .

[16] D. E. Prober, R. E. Schwall, and M. R. Beasley, "Upper critical fields and reduced dimensionality of the superconducting layered compounds," *Phys. Rev. B* **21**, 2717 (1980). http://dx.doi.org/10.1103/PhysRevB.21.2717 .

[17] H. Lei, R. Hu, E. S. Choi, J. B. Warren, and C. Petrovic, "Pauli-limited upper critical field of $Fe_{1+y}Te_{1-x}Se_x$," *Phys. Rev. B* **81**, 094518 (2010). http://dx.doi.org/10.1103/PhysRevB.81.094518 .

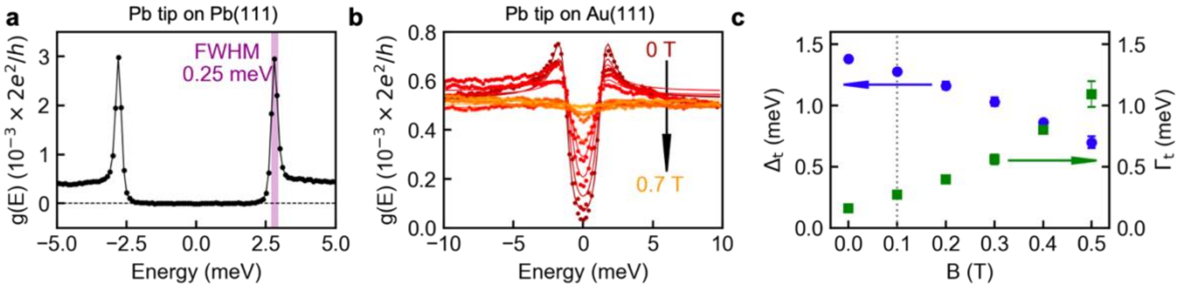

Supplementary Figure 1. Characterization of the superconducting tip. a Differential conductance spectrum after indenting the superconducting tip in a Pb(111) surface. FWHM, full-width at half-maximum. **b** Magnetic-field dependence of the differential conductance spectra (dots) of the tip in (A) on an Au (111) surface. Solid lines show the fit by Supplementary Eq. 1 to each spectrum. The magnetic field is increased from 0 T (dark red) to 0.7 T (yellow) with a 0.1 T interval. **c** Fit parameters Δ_t (left axis) and Γ (right axis) as a function of the magnetic field. Error bars stand for uncertainties extracted from the fit. The spectra at B = 0.6 T and B = 0.7 T are almost flat, yielding large error bars of Δ_t and Γ in the fit results. Setup conditions: **a**, V_{set} = 5 mV, I_{set} = 200 pA; **b**, V_{set} = 10 mV, I_{set} = 400 pA.

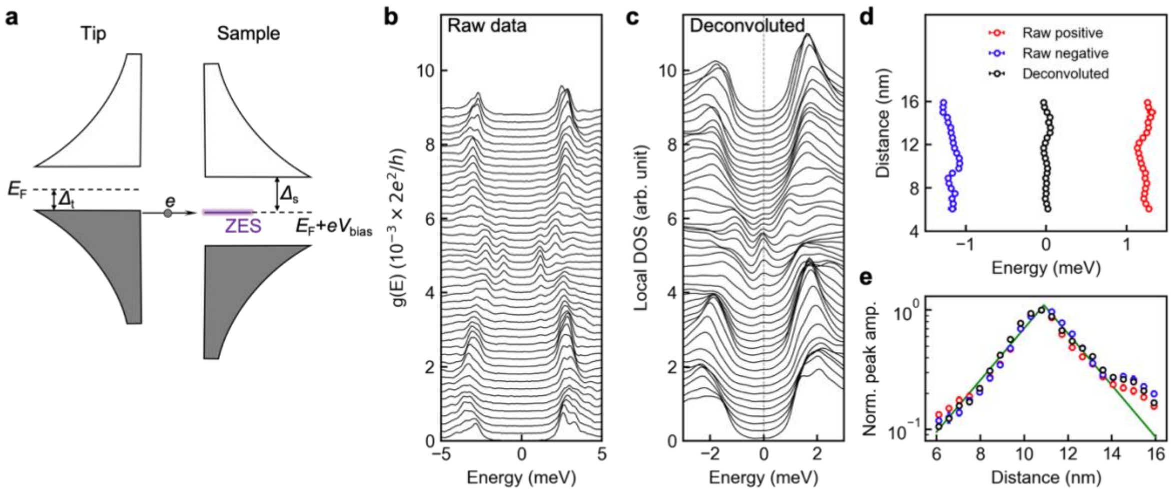

Supplementary Figure 2. Details of the structure of the zero-energy vortex bound states in $FeTe_{0.55}Se_{0.45}$. a An energy diagram showing the tunneling process from a superconducting tip to the zero-energy state (ZES) in $FeTe_{0.55}Se_{0.45}$ with a sample bias $eV_{bias} = -\Delta_t$. Both the tip and sample are superconducting with a gap size of Δ_t and Δ_s, respectively. The gray (white) area denotes the occupied (empty) states with a diverging DOS near gap edges. At this bias the diverging quasiparticle DOS leads to a maximal probability tunneling into the ZES (purple), resulting in enhanced differential conductance. E_F, Femi level. **b**,**c** Differential conductance (**b**) and corresponding deconvoluted local DOS (**c**) spectra for the line cut images shown in Figs. 3c and 3f, respectively. Spectra are shifted with a spacing of 0.2 for clarity. A Lorentzian fit is carried out to each peak inside the gap of each spectrum in **b** and **c**. **d**,**e** The peak energy (**d**) and (**e**) normalized peak amplitude (Norm. peak amp.) for points at different distances in the line cuts. The peak amplitude is normalized by the maximum of each series: red (blue) for the peak with a positive (negative) energy in **b**, and black for the peak near zero energy in **c**. The greed lines show a symmetric exponential decay around 10.9 nm (location of the vortex core center) with a decay length of 4.0 nm. Note the logarithmic scale of the vertical axis in **e**.

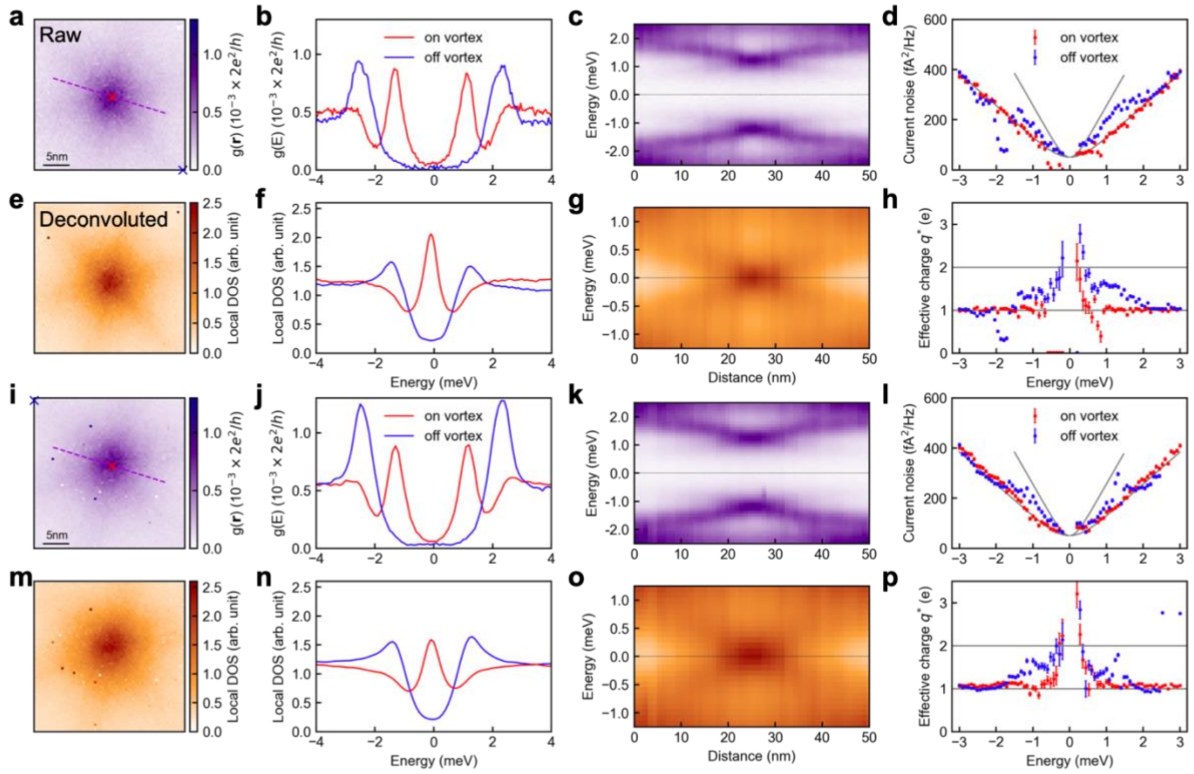

Supplementary Figure 3. Differential conductance and noise spectroscopy on different vortices in $NbSe_2$. a-g Differential conductance (**a-c** and **e-g**) and shot noise (**d**) measurements same as shown in Fig. 2 and Fig. 4a, respectively, for vortex #2 in Fig. 4b. **h**, The effective charge as a function of bias energy extracted from **d**. **i-p** Same as **a-h** for vortex #3 in Fig. 4b. Error bars in **d** and **l** are determined by the fluctuation of the current noise in time, yielding a standard deviation of 9.25 fA^2/Hz. Error bars in **h** and **p** correspond to numerical solutions of Eq. 2 in the main text, using values of upper and lower bounds indicated the error bars in **d** and **l**, respectively.

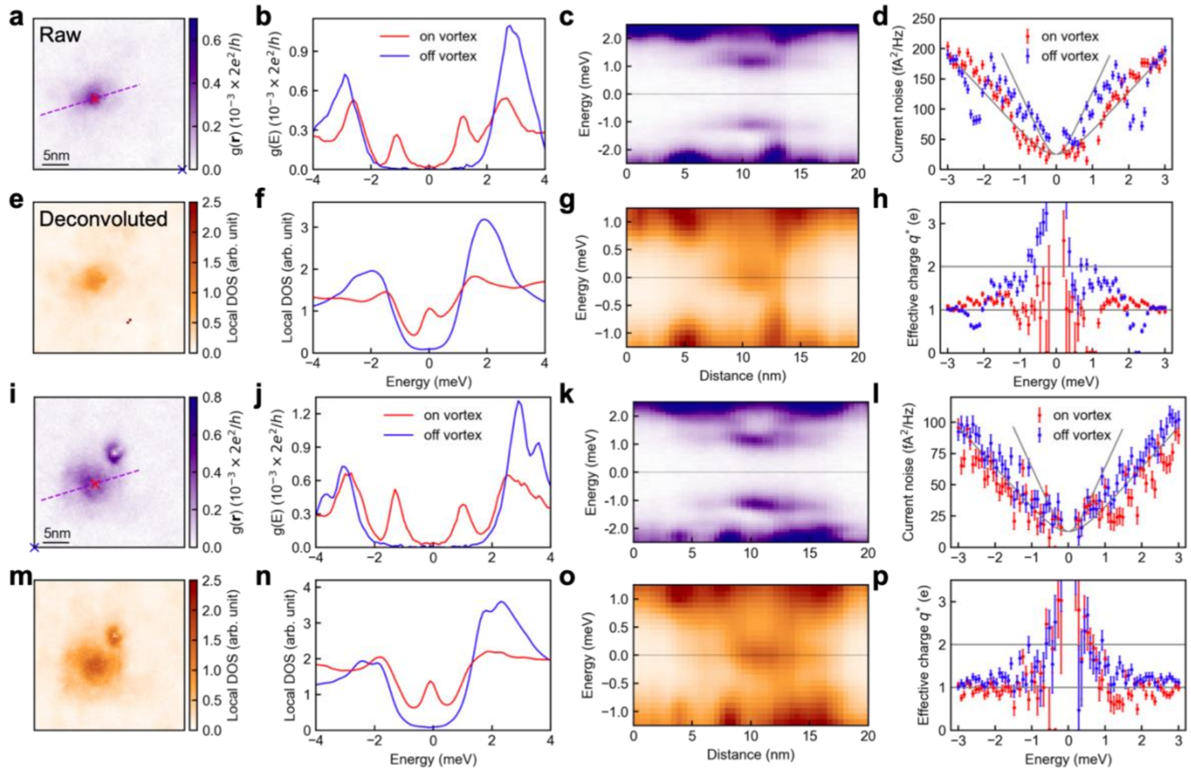

Supplementary Figure 4. Differential conductance and noise spectroscopy on different vortices in $FeTe_{0.55}Se_{0.45}$. a-g Differential conductance (**a-c** and **e-g**) and shot noise (**d**) measurements same as shown in Fig. 3 and Fig. 4c, respectively, for vortex #2 in Fig. 4d, except for a junction resistance R_J = 5 MOhm used in noise spectroscopy. **h**, Effective charge as a function of bias energy extracted from **d**. **i-p** Same as **a-h** for vortex #3 in Fig. 4d, except for a junction resistance R_J = 10 MOhm used in noise spectroscopy. Error bars in **d** and **l** are determined by the fluctuation of the current noise in time, yielding a standard deviation of 6.77 fA^2/Hz. Error bars in **h** and **p** correspond to numerical solutions of Eq. 2 in the main text, using values of upper and lower bounds indicated the error bars in **d** and **l**, respectively. Note that a YSR impurity is observed, as indicated by the small ring feature next to the vortex in **i**.

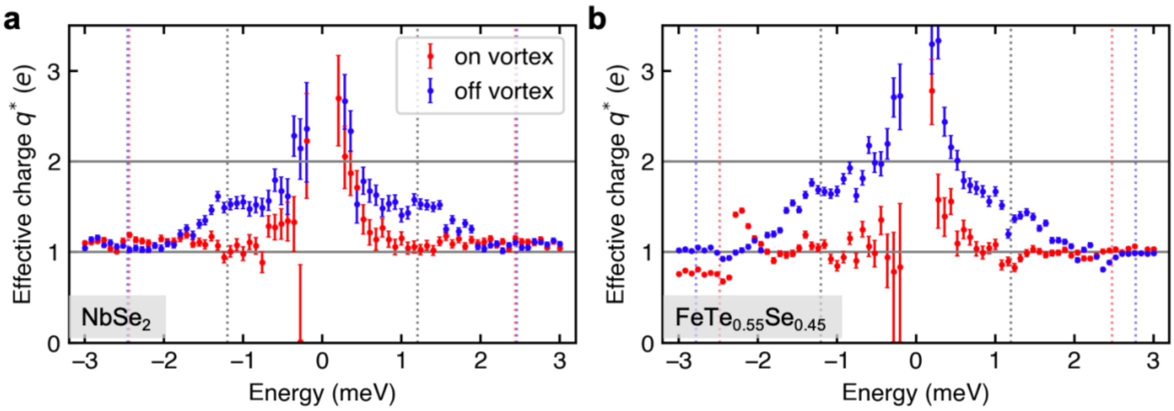

Supplementary Figure 5. Effective charge spectra on and off vortex in $NbSe_2$ and $FeTe_{0.55}Se_{0.45}$. a,b The effective charge numerically extracted from Fig. 4a and Fig. 4c, respectively, by Eq. 2 in the main text. Error bars correspond to numerical solutions using values of upper and lower bounds indicated the error bars in Fig. 4a and Fig. 4c, respectively. A further increase of q^* above within ±0.4 meV is caused by an increasing Andreev (and possible multiple Andreev) contribution because of a vanishing tip DOS. Unfortunately, the uncertainty also increases quickly for the numerical solution within this range due to the divergence of the coth function when $V_{\text{bias}} \rightarrow 0$ in Eq. 2 in the main text.

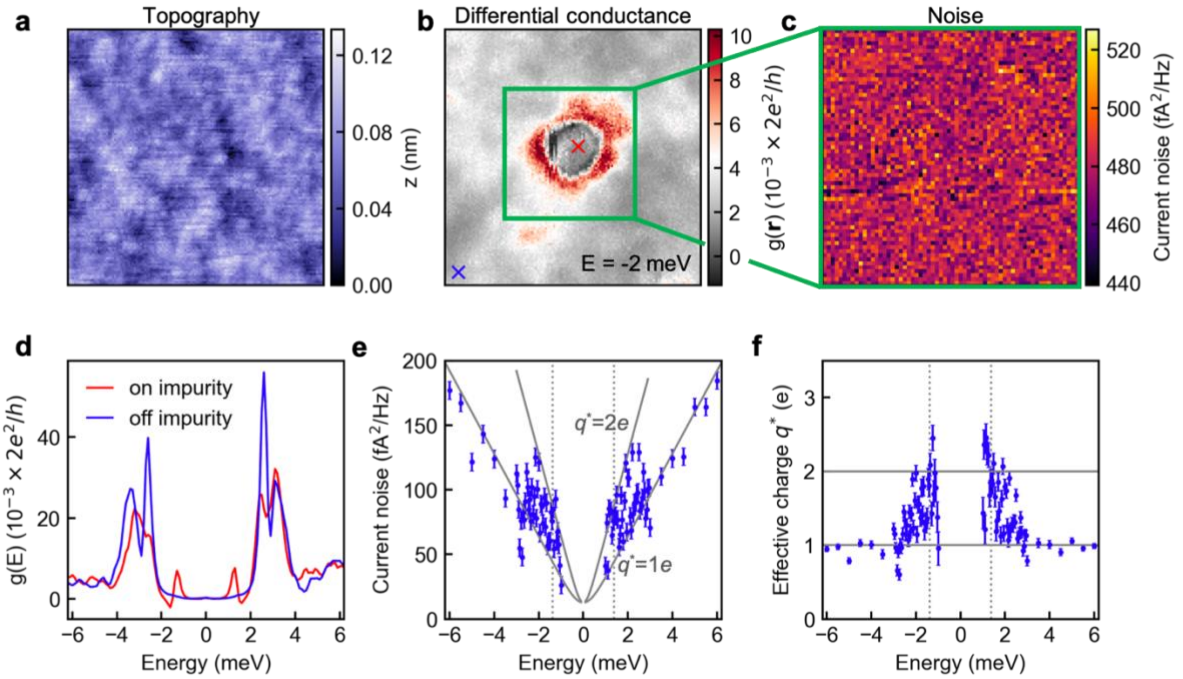

Supplementary Figure 6. Noise measurements near a YSR impurity at zero magnetic field. **a**,**b** STM topography (**a**) and differential conductance (**b**) for bias V_{bias} = -2 mV for the same field of view (20 nm × 20 nm). A ring feature with enhanced (reduced) conductance outside (inside) indicates that resonant tunneling occurs when approaching the impurity. **c** Grid spectroscopic map of noise measured near the impurity (the green square in **b**) at the same bias V_{bias} = -2 mV. The ring feature is absent in noise. **d** Differential conductance spectra measured on and off the impurity, denoted by the red and blue crosses in **b**, respectively. **e**,**f** Noise spectrum (**e**) and its corresponding effective charge (**f**) spectrum taken at off impurity position, showing a step from 1*e*- to 2*e*-noise. Error bars in **e** are determined by the fluctuation of the current noise in time, yielding a standard deviation of 6.77 fA^2/Hz. Error bars in **f** correspond to numerical solutions of Eq. 2 in the main text, using values of upper and lower bounds indicated the error bars in **e**, respectively. Setup conditions: **a**, **b**, and **d**, V_{set} = -8 mV, I_{set} = 4 nA; **c**, V_{set} = -2 mV, I_{set} = 800 pA, R_{J} = 2.5 MOhm; **e**, R_{J} = 10 MOhm.

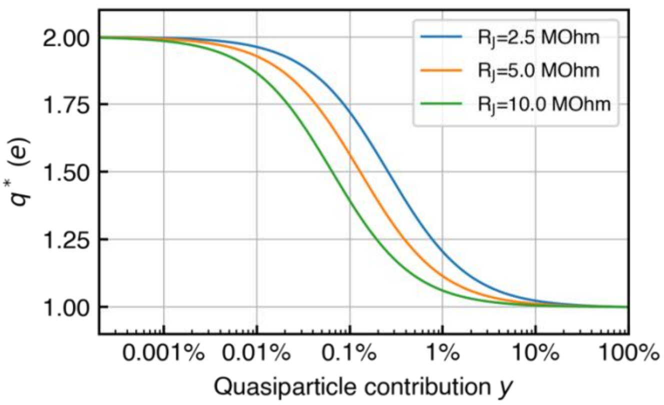

Supplementary Figure 7. Simulation of effective charge as a function of quasiparticle contribution. Three different junction resistance R_J (hence transparency) of 2.5, 5, and 10 MOhm in our experiments are used as an input in the model (see Supplementary Note 5 for details of the model).

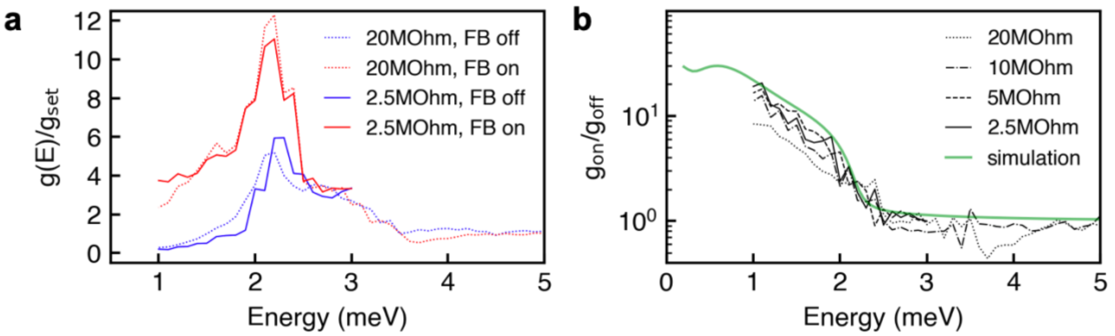

Supplementary Figure 8. Transparency of the junction with and without feedback control. a Differential conductance spectra, normalized by setup conductance g_{set}, for different junction resistance (solid line, R_J = 2.5 MOhm; dotted line, R_J = 20 MOhm). Red and blue lines correspond to spectra taken with (FB on) and without (FB off) feedback control, respectively. **b** Measured conductance ratio for different junction resistance. The green line shows the simulation result (see Supplementary Note 6), in good agreement with all the data. The small dip near zero energy originates from the difference between the energy gaps of the tip and sample. Note the logarithmic scale of the vertical axis.

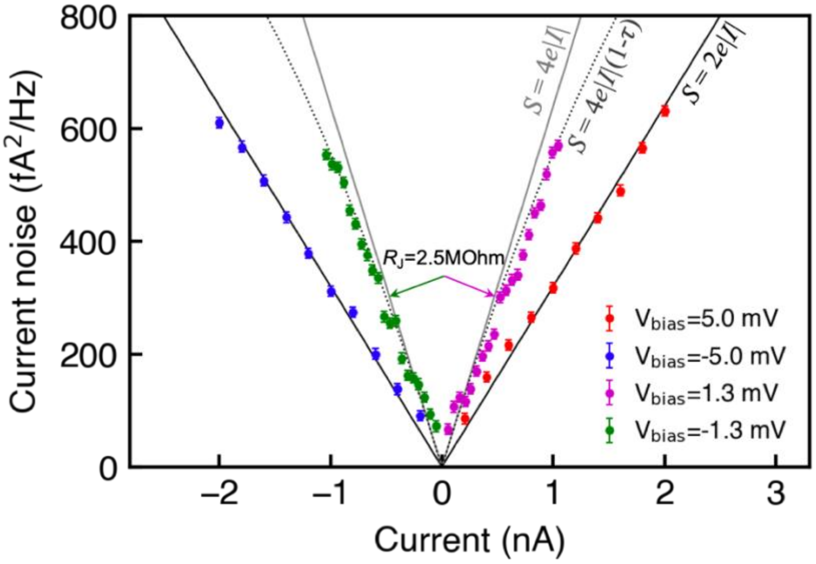

Supplementary Figure 9. Linear dependence of current noise as a function of tunneling current in the high-bias limit ($|eV_{bias}| >> k_BT$ = 0.2 meV for T = 2.3 K). Red (blue) data show current noise measured on $NbSe_2$ at a fixed positive (negative) bias eV_{bias} = ±5 meV outside the gap. Purple (green) data show current noise measured at a fixed positive (negative) bias $eV_{bias} = \pm\Delta_t$ = ±1.3 meV in the gap. Purple (green) arrows show the condition where measurements in Fig. 4 are taken. Error bars are determined by the fluctuation of the current noise in time, yielding a standard deviation of 9.25 fA^2/Hz. The lines show theoretical expectations of shot noise, for q^*=1e (black), q^*=2e (gray), and q^*=2e with correction for nonlinear junction transparency (black dotted line).

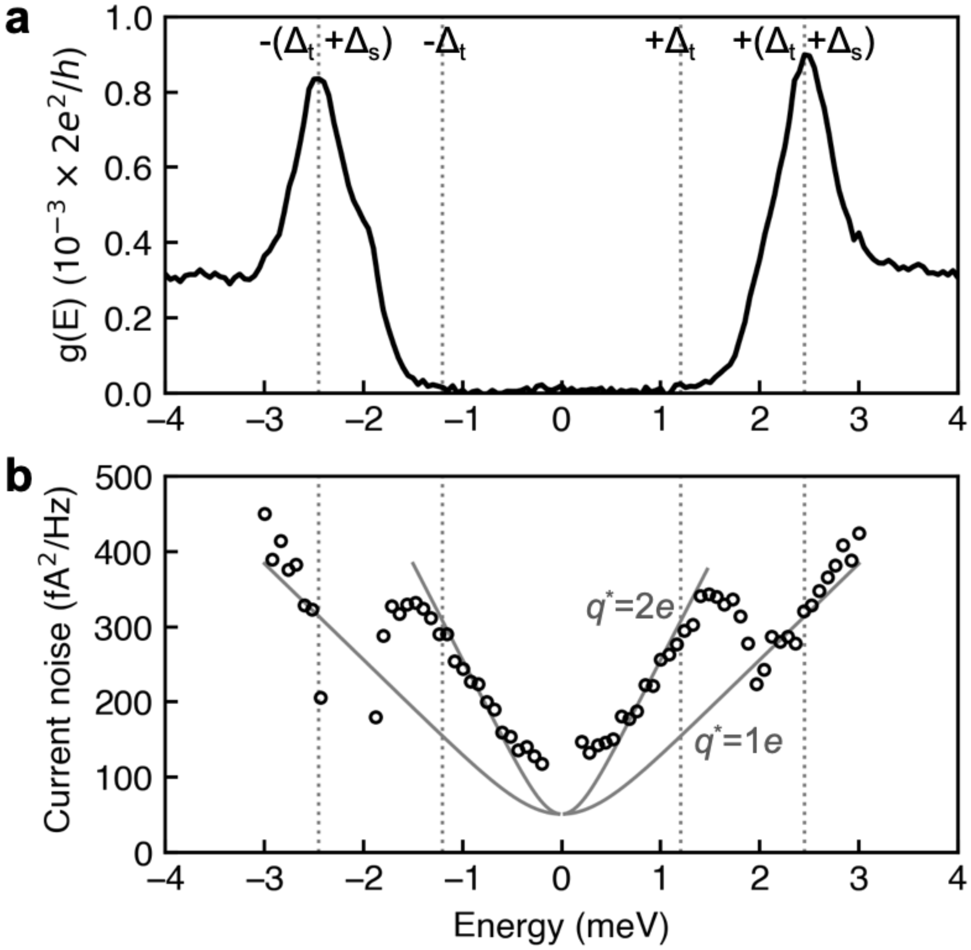

Supplementary Figure 10. Differential conductance and noise spectra measured on $NbSe_2$ at zero field. a Differential conductance spectrum and **b** corresponding noise spectrum measured at B = 0 T and a random location on a $NbSe_2$ sample. Noise data increase from q^*=1e curve starting at $\pm(\Delta_t + \Delta_s)$, towards to q^*=2e curve inside the gap. Setup conditions: **a**, V_{set} = -5 mV, I_{set} = 200 pA; **b**, R_J = 2.5 MOhm.
